# Observations of amyloid breakdown by proteases over time using scanning acoustic microscopy

**DOI:** 10.1038/s41598-023-48033-4

**Published:** 2023-11-24

**Authors:** Katsutoshi Miura, Toshihide Iwashita

**Affiliations:** Department of Regenerative and Infectious Pathology, Hamamatsu University School of Medicine, 1-20-1 Handa-Yama, Higashiku, Hamamatsu, 431-3192 Japan

**Keywords:** Biological techniques, Biophysics, Computational biology and bioinformatics, Neuroscience, Structural biology, Anatomy, Diseases, Medical research, Neurology

## Abstract

Amyloid consists of insoluble beta-fibrillar proteins with stable structures. The Congo red staining method for histologically detecting amyloid is unsuitable for quantitatively assessing amyloid fibers. Scanning acoustic microscopy (SAM) detects the attenuation of sound (AOS) through sections. This study aimed to clarify whether AOS values reflected the amount of amyloid fibril degradation in tissues. Formalin-fixed paraffin-embedded unstained sections of various types of amyloidosis were digested with different endopeptidases. The AOS images after digestion were observed over time via SAM. The corresponding Congo red-stained images were followed to identify the amyloid. The amyloid and nonamyloid portions were statistically examined over time to determine the changes in the AOS values. Most of the amyloid areas showed significantly different AOS values from nonamyloid portions before digestion and significantly decreased after digestion; these findings corresponded with the disappearance and waning of the Congo red staining in the light microscopic images. Some nonamyloid areas with high AOS masked the reduction in AOS in the amyloid areas. The method used in this study may help detect the amyloid quantity and determine the appropriate treatment method for removing amyloid deposits from tissues.

## Introduction

The increase in systemic or local amyloid deposition with age can lead to organ dysfunction^1^. The deposited amyloid is a relatively insoluble beta-fibrillar protein with stable structures that need to be broken down^2,3^. Two methods have been used to treat systemic amyloidosis: reduction of the precursor proteins and degradation of the amyloid fibrils^4^. Although amyloid fibrils are relatively resistant to enzymatic digestion, they can be almost entirely broken down by macrophages within lysosomes^5^. Amyloid degradation can be histologically determined using the Congo red staining method.

However, evaluating the decrease in the amount of amyloid is challenging because the intensity of the staining varies with the thickness of the section and the staining condition. Thus, accurate evaluation methods are mandatory to assess the decomposition of amyloid quantitatively.

Scanning acoustic microscopy (SAM) detects the attenuation of sound (AOS) through sections. Attenuation is the loss of intensity and amplitude as sound waves travel through a medium^6^. Absorption, which involves converting acoustic energy into heat, is the primary source of attenuation in soft tissue; additionally, scatter is known to contribute to AOS^7^. Short-wavelength ultrasound waves are necessary for higher resolution during histology. However, short wavelength frequencies indicate high-frequency waves, which lose more energy than low-frequency waves. The extent of attenuation depends on the type of tissue through which the sound wave travels. The intrinsic propensity of attenuation at a given frequency is represented by its attenuation coefficient (α) and measured in dB / MHz x cm. The α at 1 MHz in the bone, kidney, fat, and blood are 20, 1, 0.6, and 0.18 dB/cm, respectively^8^, indicating that the AOS increases with the density and stiffness of the tissue^9^.

We hypothesized that amyloid fibrils can reduce the AOS values based on the degree of degradation. This study aimed to evaluate the AOS values of amyloid using the same section over time in order to determine whether the values reflect the amount of degraded amyloid. This novel method of AOS observation may prove helpful in evaluating the extent of amyloid breakdown in the tissue.

## Materials and methods

### Human specimen preparation

Stored paraffin blocks containing amyloid without a link to the patient’s identity were used for this study. The study protocol conformed to the ethical guidelines of the Declaration of Helsinki and was approved by the ethical committee of the Hamamatsu University School of Medicine (approval no. 19-180). Written informed consent was obtained from all subjects. All procedures were conducted according to the guidelines and regulations of the ethical committee. All tissue samples for pathological diagnosis were fixed in a 10% buffered formalin solution, embedded in paraffin, and cut into flat sections [10-µm-thick sections were made for SAM, whereas 4-µm-thick sections were prepared for light microscopy (LM)].

### The type of amyloid

Formalin-fixed paraffin-embedded (FFPE) sections from patients with various types of amyloid including amyloid angiopathy consisting of the Aβ protein, wild-type transthyretin (TTR) amyloidosis of the heart, localized aortic valve (AV) amyloidosis with undetermined precursor proteins, immunoglobulin light chain (AL) amyloidosis, and amyloid A protein (AA) amyloidosis, were used in this study.

### Proteases

Various endopeptidases, including actinase (Funakoshi, Tokyo, Japan), collagenase type2 (Worthington, Lakewood, NJ), and neprilysin (BioLegend, San Diego, CA), were used to degrade the amyloid. Actinase and collagenase type 2 were dissolved at 1 mg/ml in phosphate buffer saline (PBS) containing 0.5 mM CaCl_2_; likewise, Neprilysin (620 pg/ml) was dissolved in PBS (pH 7.4). The enzyme solution was mounted on the section and incubated at 37 °C for 0.5, 1, and 2 h. The sections were washed in distilled water at each time point and observed under a microscope. After observation, the same section was reincubated in the enzyme solution for up to 2 h.

### SAM observations

**Figure 1:**
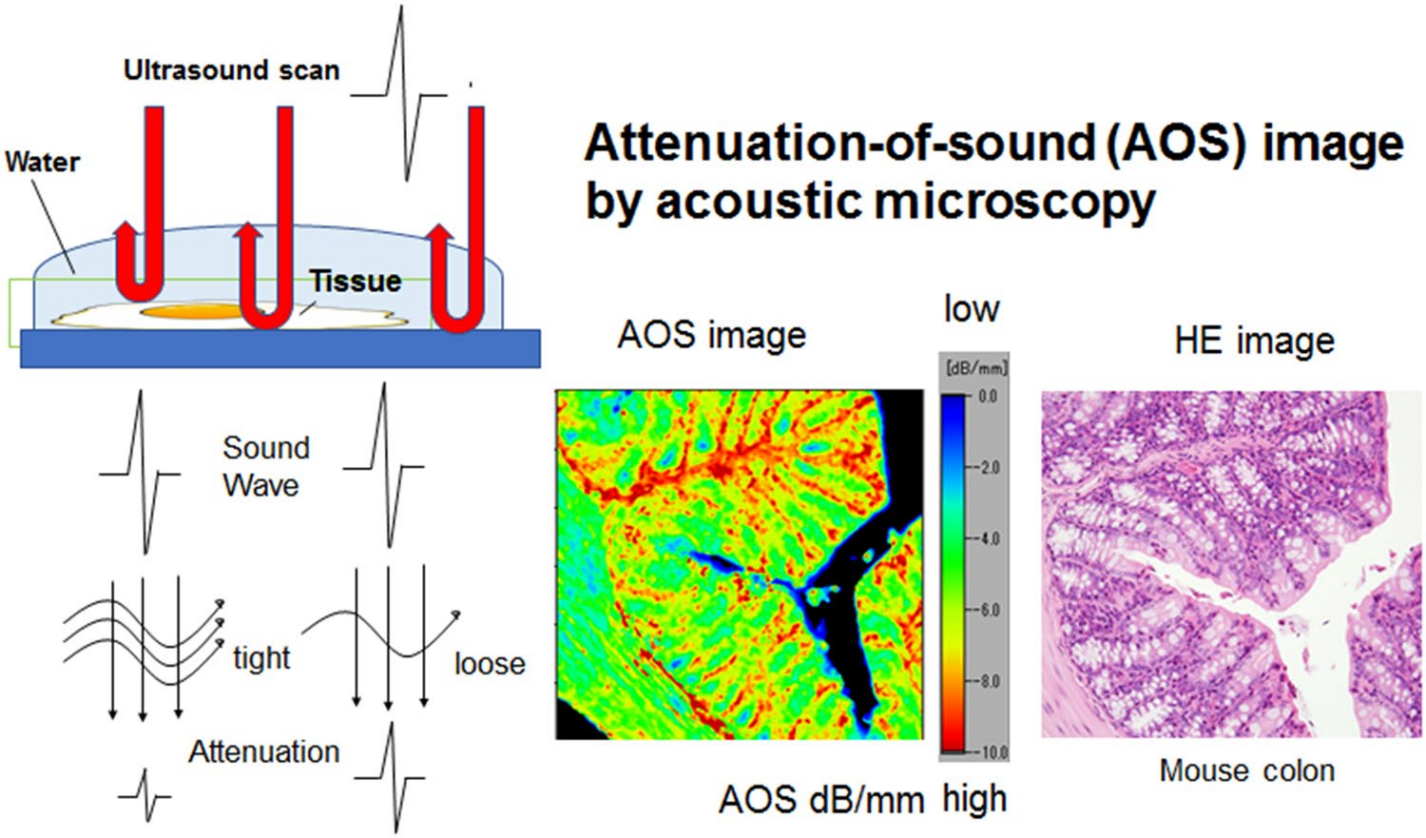
The principles of the attenuation-of-sound (AOS) image. Sound waves irradiating from the transducer hit and return from the surface and bottom of the specimen. The waves lose more energy through tight fibrous tissues than loose tissues. Plotting the attenuation values from each point on the section creates a histology image in color code (for example, from the mouse colon at the center bottom). The color bar code presents the highest attenuation area in red and the lowest attenuation area in blue, and the corresponding light microscopic image in hematoxylin and eosin staining is located at the bottom right.

The sections were examined using a SAM system (AMS-50AI; Honda Electronics, Toyohashi, Aichi, Japan) with a central frequency of 320 MHz and a lateral resolution of 3.8 µm (Fig. 1) ^10,11^. The transducer was excited with a 2-ns electrical pulse to emit an acoustic pulse ^12^. The section was placed on the stage, and distilled water was used as coupling fluid between the transducer and the section. The transducer was used for transmitting and receiving the wave signal. Waveforms reflected from the surface and the bottom of the sample were compared to measure the AOS at each point. The waveform from a glass surface without the specimen was considered the zero AOS area (black) and used as a reference. The section was scanned along the X–Y axis within 2.4 mm × 2.4 mm, 1.2 mm × 1.2 mm, and 0.6 mm × 0.6 mm square areas. It took a few minutes for a single scan. After the scan, the AOS value at each point was plotted on the screen according to color code to make an AOS image.

### LM observation

LM slides with Congo red staining in the vicinity of the SAM sections were prepared to identify the amyloid deposits observed under a polarized light microscope. After protease digestion, the sections were stained in Congo red and compared with the corresponding AOS images.

### Statistical analyses

The means and standard deviations (SD) of the AOS values were calculated from at least five areas per slide. Mean AOS values between amyloid and non-amyloid portions and mean AOS values at different time points following protease digestion were compared using student’s t-test or Welch’s t-test. A commercial statistics software (BellCurve for Excel; Social Survey Research Information, Tokyo, Japan) was used to calculate the mean areas-of-interest values, make dot blot graphs, and analyze t-tests. Before the statistical analyses, all data sets with normal distribution were compared in a test for the difference between mean values. A *p*-value of < 0.05 was considered significant.

## Results

### Comparison of the AOS values and Congo red stains

**Figure 2:**
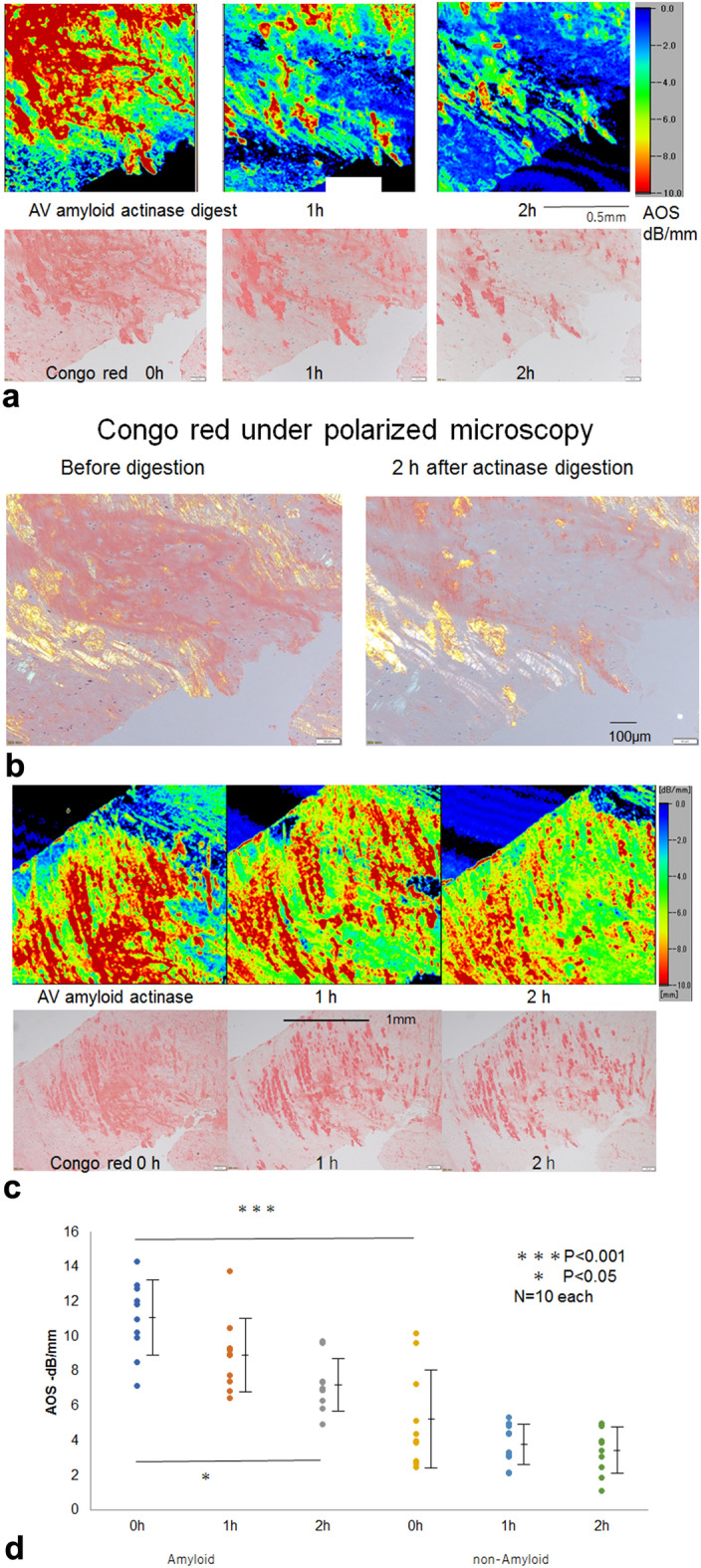
(**a**) AOS and LM images of the aortic valve after actinase digestion. The AOS values in the amyloid portions were high before digestion and decreased gradually. The corresponding L M image with Congo red staining showed a gradual disappearance of the positive areas. (**b**) Congo red staining under polarized light. Amyloid-positive areas with apple-green or apple-yellow birefringence decreased at 2 h after actinase digestion. White and striated fibers are collages where some amyloids coexist. (**c**) AOS and LM images of the aortic valve of another portion in lower magnification. Congo red-positive areas are consistent with high AOS areas and reduce simultaneously after digestion. (**d**) Dot blot of AOS values after digestion. Mean AOS values (± standard deviation) after digestion were plotted to compare amyloid and nonamyloid areas. The amyloid portions showed significantly higher AOS values than nonamyloid portions (P < 0.001). The amyloid portions were significantly reduced at 2h after digestion (*P* < 0.05), whereas the background nonamyloid areas displayed no significant reduction.

The AV amyloidosis section was digested by actinase and collagenase, and the AOS images and Congo red-positive areas were examined over time (Fig. 2a–c, Supplementary Fig. 1). The AOS in the amyloid portions displayed greater values corresponding to the Congo red-positive areas under polarized light (Fig. 2b, Supplementary Fig. 1). The AOS values in the amyloid and nonamyloid portions showed significant differences before digestion (Fig. 2d, Supplementary Fig. 2) and gradually decreased over time. The disappearance of the Congo red-positive areas well corresponded to the vanishing areas of the AOS image.

After actinase digestion, the amyloid portions showed a statistically significant difference between 0 and 2 h (*P* < 0.05), whereas the nonamyloid valve areas revealed no significant difference (Fig. 2d).

### Senile heart amyloid after collagenase digestion

**Figure 3:**
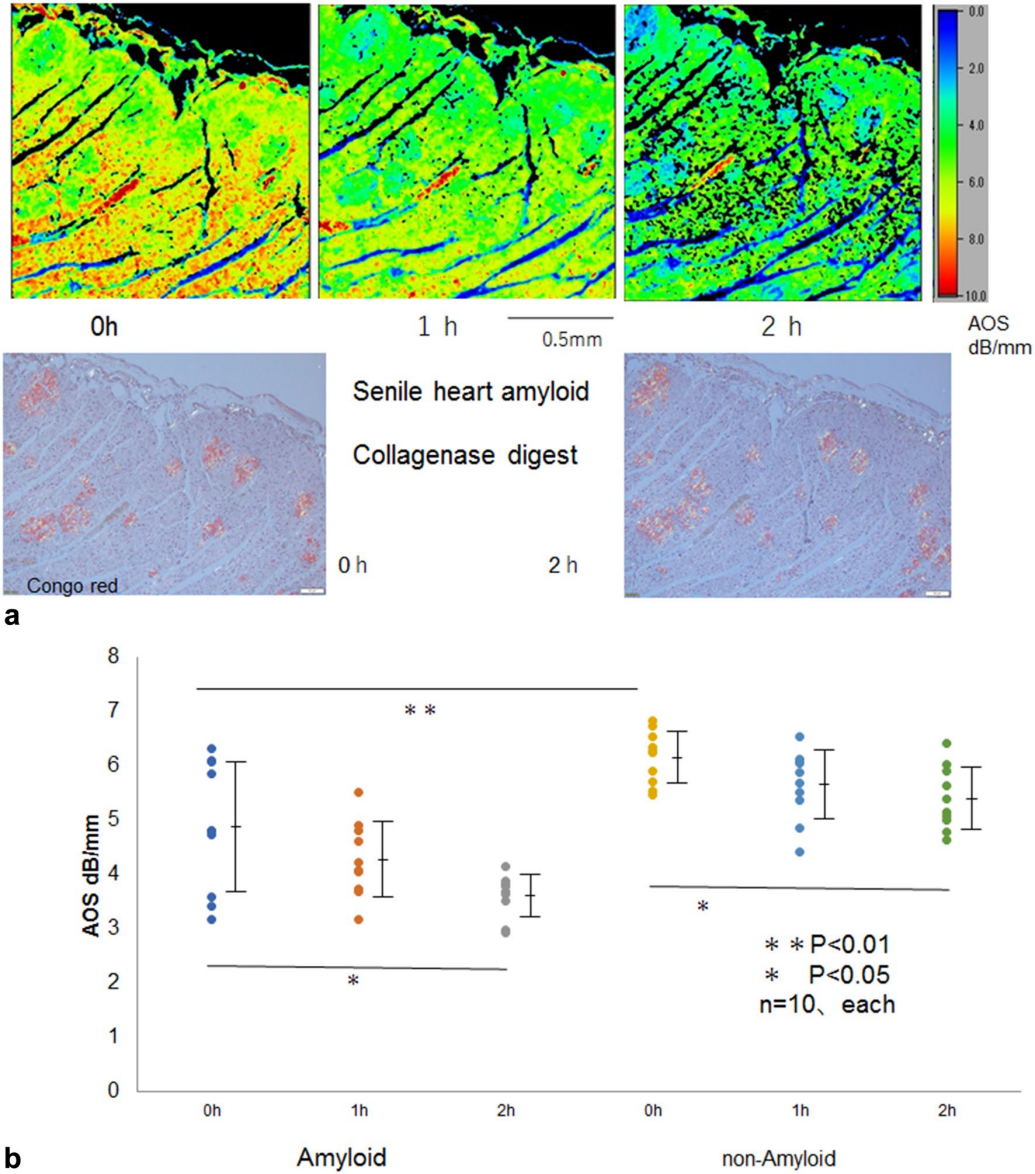
(**a**) AOS and LM images of the senile heart after collagenase digestion. Upper row: AOS image, lower left: LM image with Congo red staining under polarized light. (**b**) Dot blot of AOS values after digestion. Mean AOS values (± standard deviation) after digestion were plotted to compare amyloid and nonamyloid areas. Before digestion, the amyloid portions displayed significantly lower values than the surrounding cardiac muscles (*P* < 0.01). Both amyloid and nonamyloid areas gradually decreased AOS values and significantly reduced at 2h after digestion (*P* < 0.05, respectively).

In the AOS image, the amyloid portions were obscured in the cardiac muscles before digestion (Fig. 3a). After collagenase digestion, the amyloid portions gradually appeared as lower AOS areas than surrounding nonamyloid areas that emerged as dotted areas.

Both amyloid and nonamyloid portions showed a statistically significant decline in the average AOS values at 2 h after digestion (P < 0.05) (Fig. 3b).

### Senile lung amyloid after collagenase digestion

Amyloid deposition was seen along the alveolar walls (Supplementary Fig. 3) and showed significantly different AOS values than nonamyloid portions (Supplementary Fig. 4). The AOS values of both amyloid and nonamyloid portions gradually decreased over time after collagenase digestion. A significant decrease was observed in both portions at 2 h after digestion.

### Amyloid angiopathy after neprilysin digestion

**Figure 4:**
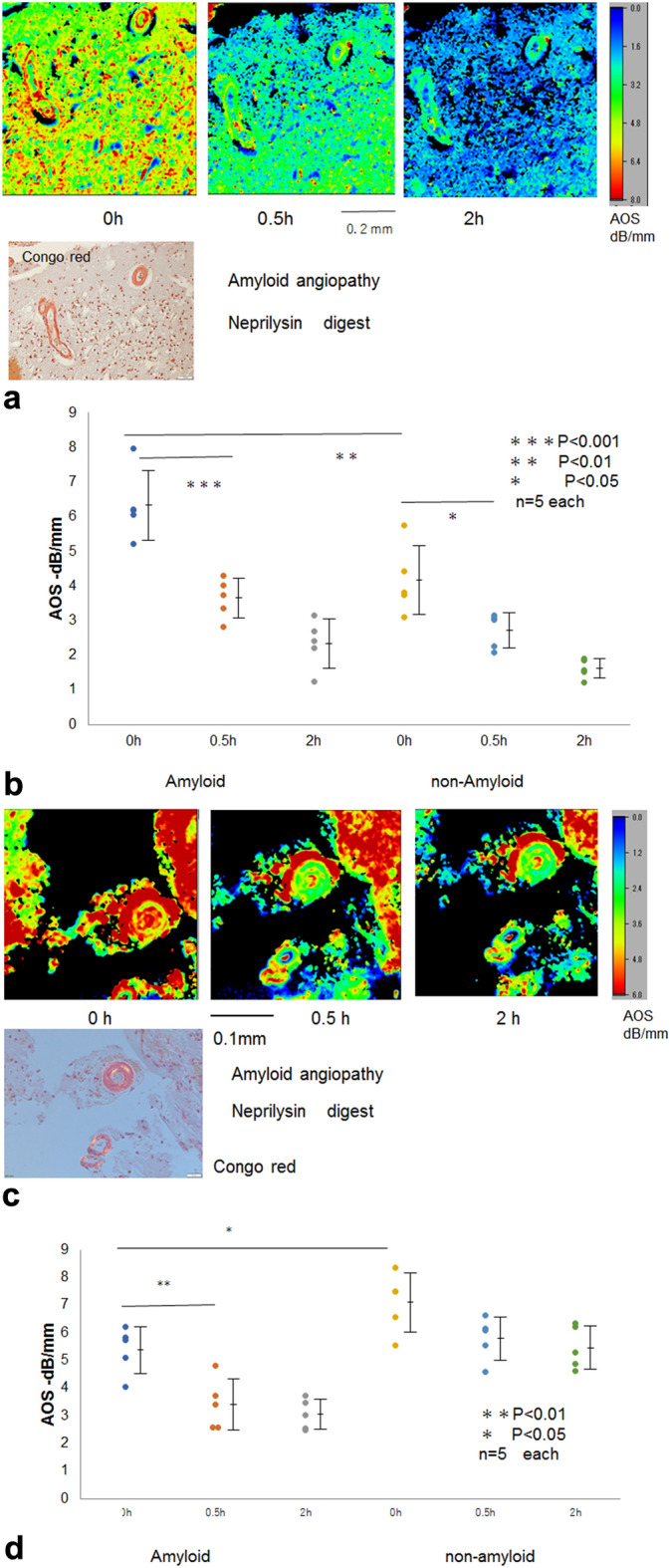
Neprilysin digestion in amyloid angiopathy. (**a**) Case A, wherein beta proteins were deposited as amyloid on the vascular walls. Amyloid areas showed significantly high AOS values compared with the surrounding brain tissues. Upper row: AOS image, lower left: LM image with Congo red staining. (**b**) Dot blot of AOS values after digestion. Mean AOS values (± standard deviation) after digestion were plotted to compare amyloid and nonamyloid areas. AOS values of both areas significantly decreased at 1h after digestion (*P* < 0.001 and *P* < 0.05, respectively). (**c**) Case B, wherein amyloid deposits on the vascular walls were positively stained with Congo red. Upper row: AOS image, lower left: LM image with Congo red staining under polarized light. (**d**) Dot blot of AOS values after digestion. Mean AOS values (± standard deviation) after digestion were plotted to compare amyloid and nonamyloid areas. The AOS values between amyloid and nonamyloid portions presented significant differences before digestion (*P* < 0.05). The AOS values of amyloid portions only showed a significant reduction at 1h after digestion.

Amyloid deposits on the vascular walls of two cases (A, B) showed significantly different AOS values compared with nonamyloid portions (Fig. 4a–d) and a significant reduction at 1 h after digestion with neprilysin (*P* < 0.01). The nonamyloid brain tissues of only case A demonstrated a significant decrease in AOS values at 1 h after digestion (*P* < 0.05).

### AL amyloidosis after collagenase digestion

**Figure 5:**
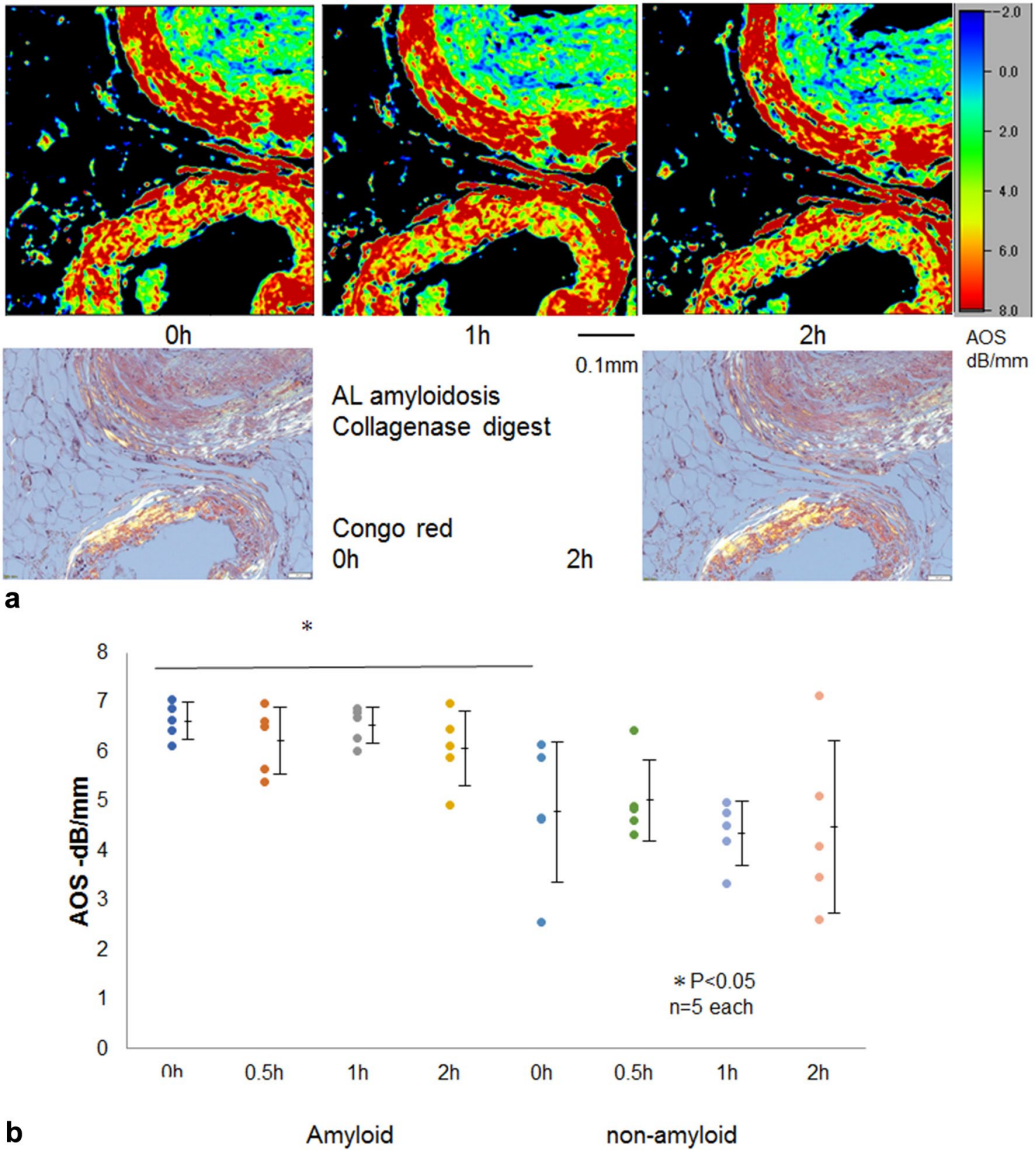
Collagenase digestion of the vascular AL amyloid. (**a**) AL amyloid was deposited on the vascular walls, which were positive for Congo red. Upper row: AOS image, lower left: LM image with Congo red staining under polarized light. (**b**) Dot blot of AOS values after digestion. Mean AOS values (± standard deviation) after digestion were plotted to compare amyloid and nonamyloid areas. The AOS values on the amyloid part showed a significant difference from the nonamyloid part (*P* < 0.05). The AOS of both amyloid and nonamyloid areas presented no remarkable reduction after digestion.

Amyloid is deposited on the vascular walls in patients with systemic AL amyloidosis. We tried to digest amyloid sections with collagenase, but the AOS and LM images with the Congo red stains showed unremarkable changes over time (Fig. 5a). No significant difference was found in AOS values between the amyloid and nonamyloid portions (Fig. 5b).

No significant decline in the average AOS values was observed in the amyloid and nonamyloid portions after digestion.

### Collagenase digestion of vascular AA amyloidosis

**Figure 6:**
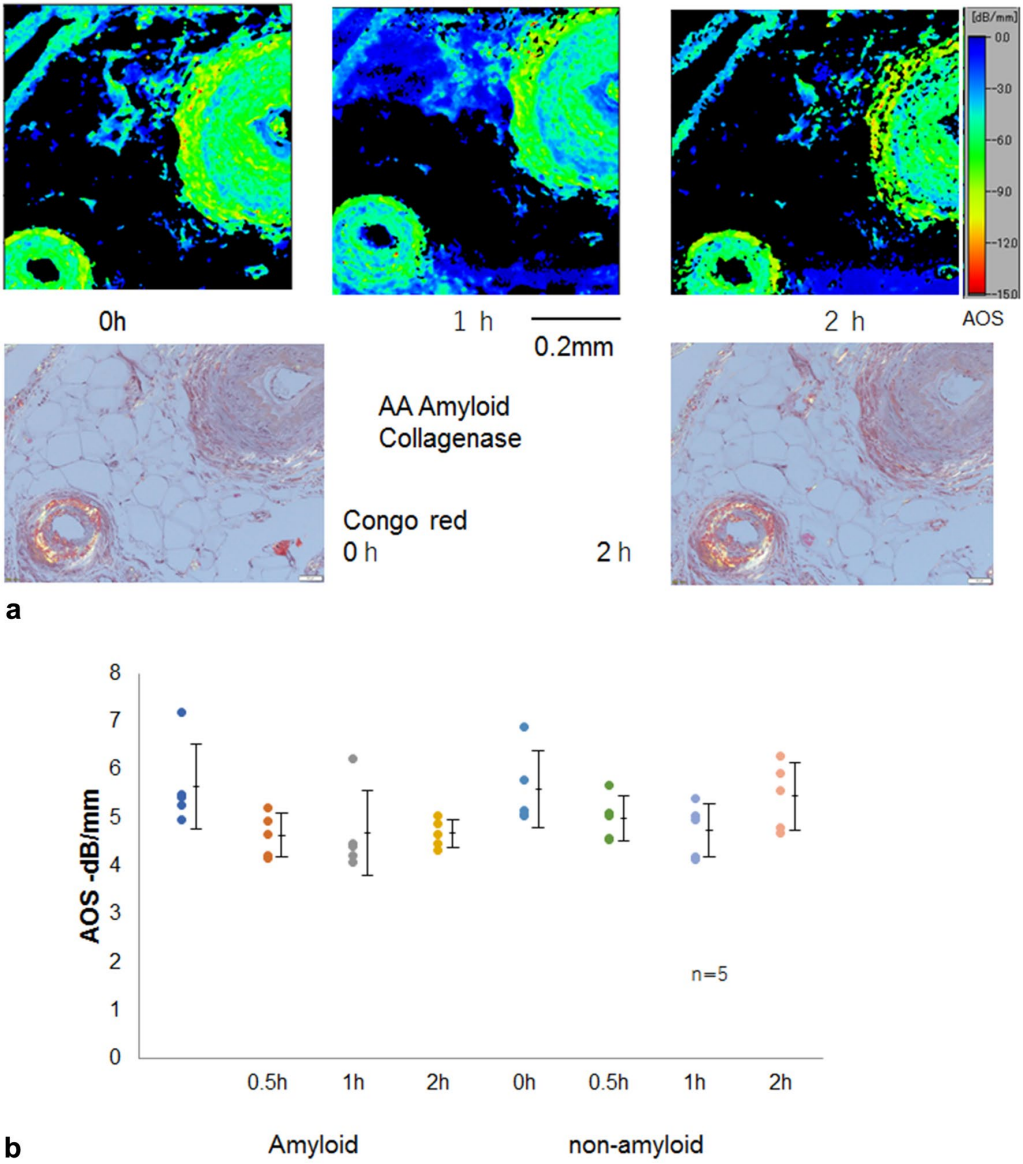
Collagenase digestion of vascular AA amyloid. (**a**) The Congo red staining shows AA amyloid deposited on the vascular walls. Upper row, AOS image; lower left, LM image with Congo red staining under polarized light. (**b**) Dot blot of AOS values after digestion. Mean AOS values (± standard deviation) after digestion were plotted to compare amyloid and nonamyloid areas. Amyloid and nonamyloid portions of vascular walls showed no significant difference in AOS values. The AOS values of both amyloid and nonamyloid parts exhibited no significant reductions after digestion.

The AOS images of amyloid and nonamyloid areas in the vascular walls showed no remarkable difference (Fig. 6a, b). Both areas showed a little gradual reduction in AOS values. However, no significant decreases in AOS values were observed over time.

## Discussion

In the AOS image, amyloid and nonamyloid portions exhibited significantly different values. Most amyloid parts presented higher AOS values than the surrounding nonamyloid parts, but some amyloids had lower AOS values (Figs. 3b, 4d, and Supplementary Fig. 4), because nonamyloid parts had greater AOS values with dense fibrous structures, such as vascular walls and dense connective tissues. AOS images corresponded with Congo red staining image, so it was easy to follow amyloid breakdown over time using AOS images.

The degradation process of amyloid fibers by enzymes was statistically evaluated over time via AOS values.

The ease of amyloid degradation differed depending on the type of amyloid, enzyme, or organ. The reduction in AOS in the amyloid portion was difficult to detect in cases where the AOS values were high in the background nonamyloid tissues, such as in the AL amyloidosis (Fig. 5a). On the contrary, AOS decrease was readily seen in cases with low backgrounds, such as the AV and brain amyloidosis (Figs. 2a, c, and 4a). Protein degradation treatment is ideal when only the amyloid portions are affected without background damage.

The method used for protease digestion on the FFPE sections has been reported previously for antigen retrieval treatment^13^. Formaldehyde forms methylene bridges between proteins, which can hinder epitope recognition by primary antibodies. Partial digestion of the proteins unmasks the antigenic epitopes. Compared to antigen retrieval purposes, the present condition for amyloid degradation is more severe using higher dosage and longer incubation time. After digestion, the section retained its original structure for observation.

AOS observations using an ultrasonic microscope to evaluate protein degradation have several advantages (Table 1). First, it is easy to contrast with the tissue images. The attenuation image corresponds to the LM image stained with Congo red; hence, the location of the amyloid can be accurately grasped. Second, objective statistical comparison is possible owing to the numerical form of the attenuation value. Third, the same section can be used to observe the degradation process over time visually. Fourth, the enzymes applied to the sections can be selected, thereby making it possible to search for one that retains the background structure and specifically recognizes the target protein.

**Table 1:** Comparison of the two methods used to detect amyloid for breakdown.

	SAM	LM
Principle	AOS decrease	Congo red stain
Staining	Unnecessary	Necessary
Number of sections	One section	Many sections
Thickness	10 μm	Variable
Observation over time	Easy	Difficult

*SAM* scanning acoustic microscopy, *LM* light microscopy, *AOS* attenuation of sound.

Alternatively, there are some disadvantages to using ultrasound microscopy. First, the material (or section), which is affected by the fixation and specimen preparation processes, may differ from the nature of the raw protein. Notably, the properties may change from those of the original protein when affected by cross-linking between proteins, organic solvents, acids, and alkalis. Unfixed frozen sections can be used for the evaluations, but it is not easy to obtain 10-μm-thick flat sections. Moreover, FFPE sections are readily available from stored paraffin blocks. Secondly, the observation area for a single scan is limited, with a maximum of 4.8^2^ mm using our ultrasonic microscope. The third point is that the resolution depends on the transducer's performance. Although the resolution is directly proportional to the frequency, the available depth of observation becomes shorter due to the increase in the sound attenuation. A low-frequency transducer can visualize thicker sections but cannot provide high-resolution images. Additionally, acquiring the skills for manual operation takes time. The distance between the transducer and the section should be adjusted to detect pulse waves from the glass slide and the surface of the section.

Amyloidoses comprise heterogeneous disorders characterized by the deposition of abnormally folded proteins in tissues^14,15^. Amyloid deposits are formed from soluble proteins that undergo misfolding and aggregate into insoluble fibrils, leading to progressive organ damage.

The activation of enzymes that degrade amyloid fibrils can be used to treat amyloidosis. Several studies have reported the breakdown of amyloid by proteases, such as trypsin for lysozyme amyloid^16^ and MMP-1 for AA amyloid^17^. Recently, the brain beta-amyloid has been gaining popularity among aged populations and causing social and economic problems; beta-amyloid was reportedly digested by neprilysin^18,19^ and insulin-degrading enzyme^20^.

Various endopeptidases were used to digest amyloid in this study. It is important to note that the effectiveness of enzyme-based approaches may depend on the targeted amyloid fibrils and the conditions under which the enzyme is used. In addition, the safety of using enzymes must be considered to avoid tissue damage. Endopeptidases have specific cleavage sites to cut amyloid proteins, and care must be taken to minimize the impact on surrounding proteins.

Neprilysin is a zinc metalloendopeptidase with relatively broad substrate specificity^21^. The enzyme is localized to the plasma membrane of cells, where it can function to degrade extracellular peptides. Structural studies show that neprilysin preferentially cleaves peptides on the amino side of the hydrophobic amino acids. Neprilysin has been implicated in the catabolism of the amyloid beta peptides in the brain^20^.

Previous studies on the degradation of amyloid used purified amyloid fibrils instead of deposited tissues^16,22,23^. However, real-deposited tissues containing amyloid and surrounding extracellular materials were used in the current study to evaluate the specific digestion of amyloid and assess the damage to surrounding tissues.

This study has several limitations. First, amyloid degradation by enzymes was not compared between FFPE and fresh frozen sections. Fresh frozen tissues are more vulnerable to enzymatic digestion, causing more tissue damage. Second, more samples with different types of amyloid and organs are necessary to investigate amyloid breakdown. Third, more precise observation using a higher frequency transducer of SAM and electron microscopy can show the amyloid degradation process at a higher resolution.

The definitive diagnosis and the identification of the specific type of amyloid depends on the biopsy sample. Biopsy samples are generally available for enzymatic degradation. We believe that the method described in the present study will aid in finding the proper treatment for removing amyloid deposits from the tissue.

### Supplementary Information

Supplementary Figures.

## Data Availability

The data supporting this study’s findings are available from the corresponding author upon reasonable request.
