# Supplementary material for: Observations of amyloid breakdown by proteases over time using scanning acoustic microscopy
Source: Sci Rep. 2023 Nov 24;13:20642. doi: 10.1038/s41598-023-48033-4 (PMC10673902; doi:10.1038/s41598-023-48033-4)

Observations of amyloid breakdown by proteases over time using scanning acoustic microscopy

Katsutoshi Miura[1*], Toshihide Iwashita[1]

[1]Department of Regenerative & Infectious Pathology, Hamamatsu University School of Medicine, Japan

Supplementary figures

Supplementary Figure S1. Collagenase digestion of the aortic valve amyloid. Amyloid was widely deposited on the aortic valve before digestion. After digestion, the AOS values decreased over time, corresponding to Congo red staining. Upper row, AOS image; lower row, LM image with Congo red staining under polarized light

Supplementary Figure S2. Dot blot of AOS values of the aortic valve. Mean AOS values (± standard deviation) after digestion were plotted to compare amyloid and nonamyloid areas. The amyloid part exhibited significantly greater AOS values than the nonamyloid part ($P < 0.01$). Both showed significant reductions in AOS values at 1 h after digestion ($P < 0.01$).

Supplementary Figure S3. Collagenase digestion of senile lung amyloids. Amyloid was deposited along the alveolar walls. In the AOS image, vascular walls and peribronchial connective tissues had greater values, whereas perialveolar amyloid showed lower AOS values before

digestion. All parts showed a gradual reduction in AOS over time after digestion.

Upper row, AOS image; lower row, LM image with Congo red staining under polarized light

Supplementary Figure S4. Dot blot of AOS values of senile lungs. Mean AOS values (± standard deviation) after digestion were plotted to compare amyloid and nonamyloid areas. Amyloid parts showed significantly lower AOS values than nonamyloid parts ($P < 0.01$). Amyloid and nonamyloid parts exhibited significant reductions in AOS at 2 h after digestion ($P<0.01$).

Supplementary Figure 1

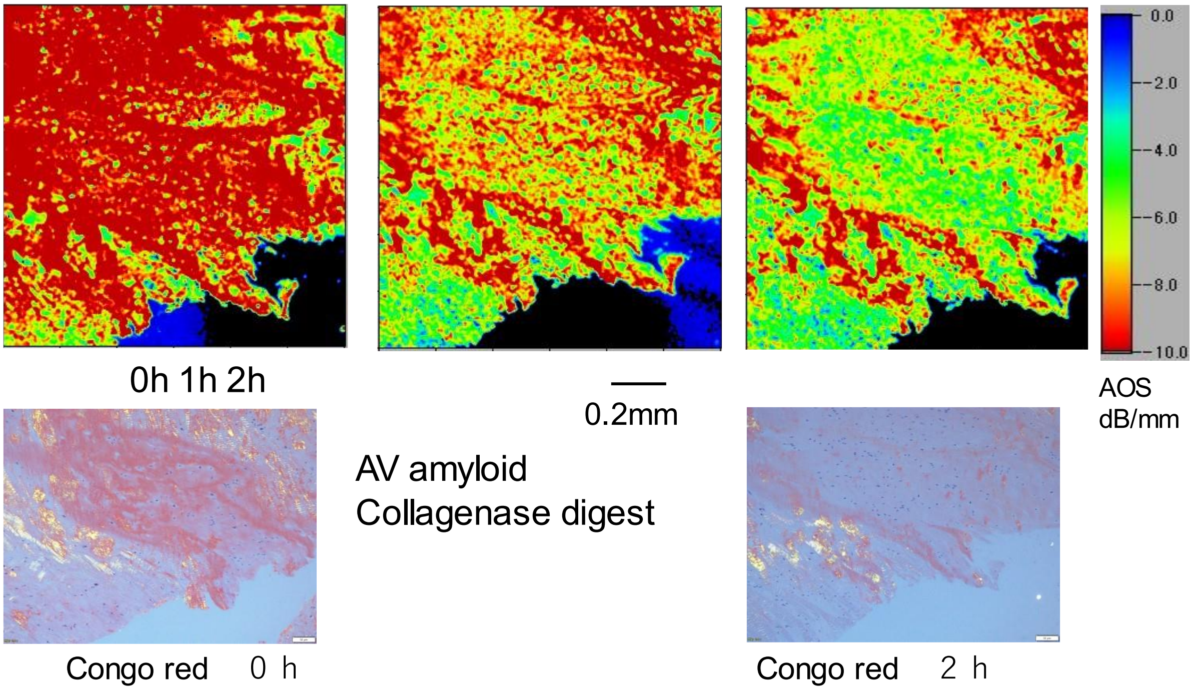

Supplementary Figure 2

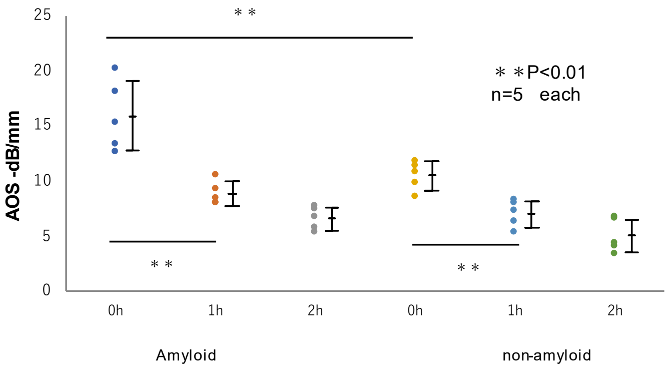

Supplementary Figure 3

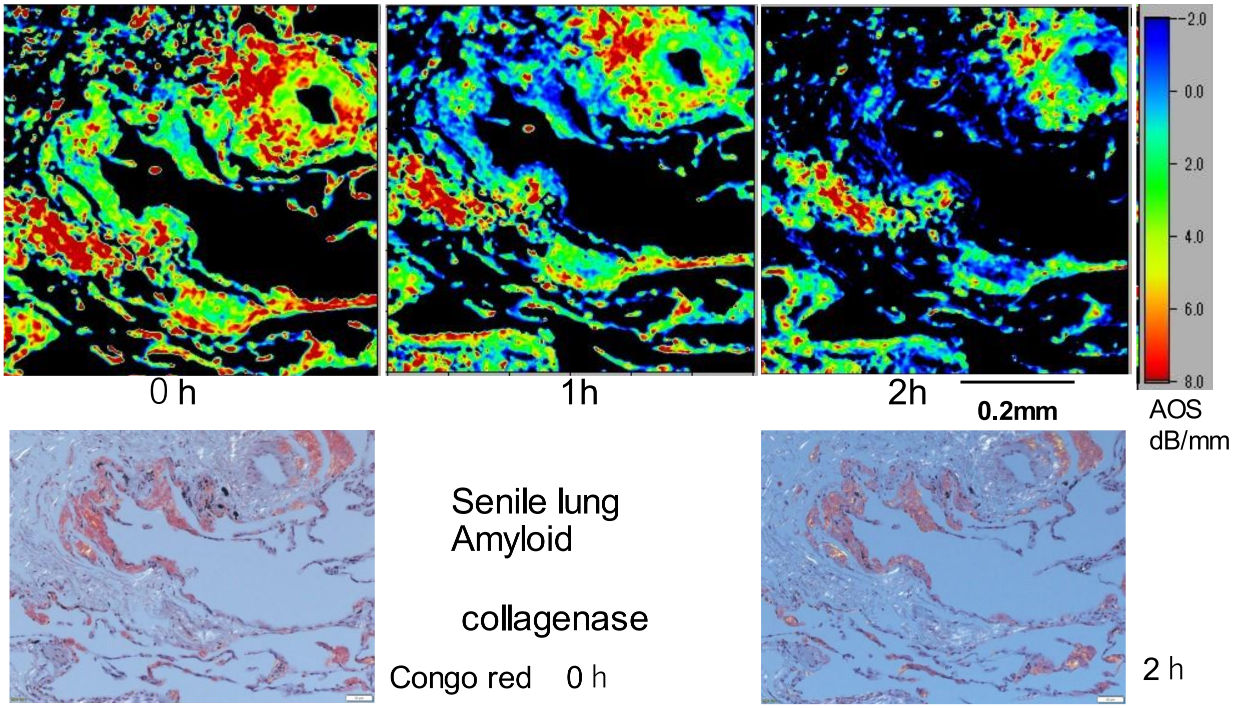

Supplementary Figure 4

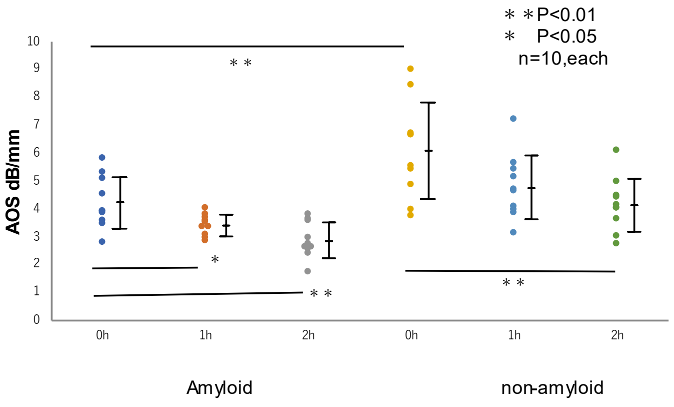

Supplement: Supplementary file 1 — Supplementary Figures. [file 41598_2023_48033_MOESM1_ESM.pdf]
